# Oxford Conference, 1913

**Published:** 1913-07-12

**Authors:** 


### THE BRITISH HOSPITALS ASSOCIATION.

### OXFORD CONFERENCE, 1913 (*continued*).

At the Session on Friday morning, June 27, Sir William Osler, President, in the chair, the following paper was read:—

### The Upkeep of Hospitals.

By KEITH D. YOUNG, Esq., F.R.I.B.A.

The upkeep of hospitals is a subject that must be considered not merely as affecting the repair and maintenance of an existing structure, but also in relation to original planning and construction. For the annual cost of maintaining a hospital can be, and is, seriously affected by mistakes in planning, by errors of construction, and by the use of inappropriate materials.

Perhaps the most important point in regard to planning is the arrangement of corridors and intercommunication generally. Two vital points to be observed in laying out the lines of communication in a hospital are: (1) That the length of corridors should be reduced to an absolute minimum; and (2) that the routes to all departments to which patients have to be directed should be as simple and obvious as possible. All excessive length of corridors affects the cost of upkeep by increasing the cost of daily cleaning, of annual cleaning, of periodical painting and whitewashing, and, if closed in, of warming. If, as in some recent examples, the corridors are open at the side, the cost of cleaning is at least doubled. Also it affects the cost of distribution of food, and adds unnecessarily to the time occupied in supervision. To illustrate this point, let me take St. Thomas's Hospital as it is, and as it might have been on a different site. The hospital is built on a long, narrow site bounded on the west by the River Thames, and on the east by Lambeth Palace Road; and the ward pavilions are placed on the west side of and at right angles to a corridor 930 feet long, which runs north and south. If it had been possible to have arranged the pavilions on each side of the corridor, the corridor could have been reduced to a little over half its existing length. When it is realised that the area of this corridor on the ground floor only is rather over one-third of an acre, the importance of the point becomes apparent.

The necessity for simplifying routes to departments to which patients have to be directed is important as vitally affecting the work of the porters, who have to handle large numbers of patients.

A most important point in the planning of a hospital is the centralising of the kitchen offices and stores, so that food for the various departments can be distributed as rapidly and with as little labour as possible.

Another very essential point is the provision of a sufficient number of coal stores all accessible to carts, so that coal can be distributed with a minimum of labour.

In these days of open-air treatment, the provision of wide balconies, on to which patients can be wheeled in their beds, is a necessity; where they do not exist the patients have to be carried by porters in stretchers into the garden—an expenditure of labour which is obviated by the use of balconies.

Details of construction affect the question of upkeep in a material way. The daily cleaning, which forms so important a part of the work of a hospital, can be facilitated, and the expense reduced by the adoption of glazed brick or tile surfaces wherever possible; and this applies not only to the daily cleaning, but to the annual cleaning and the periodical renewing which becomes necessary when painted surfaces are employed. The constant cleaning of painted work involves deterioration of the surface which must eventually be renewed, a process which requires the aid of skilled labour. On the other hand, glazed bricks or tiles can be constantly cleaned without in any way affecting the surface, and their life is unlimited.

The increasing use of hard wood doors without panels, but presenting one unbroken flush surface, for wards and their offices, involves somewhat greater cost at the outset, but is an undoubted economy in the long run. The process of cleaning is reduced to its lowest point, and the renewal of the polished surface involves no skilled labour.

The cleaning of windows is a process that can be materially simplified and cheapened by constructing them in such a way that every part is accessible without the aid of ladders. There are several kinds of reversible sashes on the market; and, while I do not intend to recommend any special make, I would point out that in choosing an apparatus, simplicity is perhaps the most important quality to be sought for. In this, as in many other things of a like nature, working parts should be as far as possible foolproof.

Periodical painting cannot, however, be abolished entirely; all woodwork and ironwork which is exposed to the weather must be repainted when necessary, or the materials will deteriorate. It is essential that all this class of work should be inspected regularly once a year, and necessary repairs carried out. It is by no means necessary that the whole of the outside paint work should be renewed every three years or at any fixed time without regard to its actual condition. Woodwork, for example, that is not exposed to the direct rays of the sun, will, if thoroughly painted in the first instance, last much longer than that which is more exposed, and it is often a wise economy to paint the sills only at more frequent intervals than those parts of the windows which are less exposed than the sills.

The importance of annual inspection applies not only to work which requires painting, but to every part of the building; and much expenditure in the matter of repairs to roofs, the pointing of chimney stacks and parapets, will be saved if small defects are taken in time.

The question of floor surfaces for wards in relation to upkeep is, I confess, a difficult one. The old-fashioned deal boarded floor, which had to be scrubbed every day, has been universally condemned and has been replaced by polished teak, terrazzo, linoleum on concrete, or the various kinds of plastic floor. The first cost of teak is considerable, and the labour involved in wax polishing great; the joints also cannot be eliminated. Terrazzo is liable to cracks, for which no sure method of prevention has yet been devised; linoleum on concrete has been largely used, and makes a satisfactory surface; but here again joints must occur; the great point in favour of a plastic floor is that it is jointless; but we do not seem yet to have arrived at a material that will keep its colour and not present an unsightly and patchy appearance. What is wanted, and what we have not yet, is a jointless floor that can be daily cleaned with a wet cloth, and whose surface does not need frequently applied skilled labour for its renewal. For the staff quarters linoleum on concrete is probably the best and most lasting floor surface available. The linoleum should be of the best quality, and should be wax polished. Treated thus, it will last for many years.

A minor point, but one quite worthy of attention, is the reduction of brasswork in order to lessen cleaning. It is quite useless to point out that well lacquered brasswork will last for years if cleaned with a dry cloth. Sooner or later it is quite certain to be attacked with some patent cleaning compound, which speedily removes the lacquer, and then the labour of cleaning is a matter of daily routine. No substitute for brass taps and valves is as yet available, but I am in hopes that some day it will be possible to make these successfully with a coating of hard white enamel.

Coming now to the engineering department, the requirements as to boilers will, of course, vary considerably with the size of the hospital. In these days the work of sterilising has grown to so great an extent that a steam boiler has become a necessity in all but the smallest class of hospital. And when steam is to be installed there can be, I think, little doubt that every possible use should be made of it. The whole of the water, both for warming and for supplies to baths, sinks, and lavatories, should be heated by steam; live steam will, of course, be used for cooking, for sterilising, for disinfecting, and for heating steam kettles and plate-warmers in ward kitchens. As to the last two points, Mr. Carnt, the general superintendent of the Manchester Royal Infirmary, tells me that by substituting steam for these purposes he has effected a saving of £350 per annum in gas without increasing the coal bill.

It is hardly necessary to point out that in installing steam boilers the work should be of the very best obtainable, and a very ample margin should be left, so that in times of greatest pressure the boilers will do the work required without overdriving.

All condensed steam should be taken back to the boiler-house to be used as feed-water to the boilers; and, as it will generally be necessary to supplement this, the water used to feed the boilers should, wherever the physical conditions require, be passed through a softening apparatus.

Wherever the amount of steam is large enough to justify the expense, an efficient economiser should be placed between the boilers and the chimney shaft. By this means a large proportion of the hot gases from the boiler flues is used to heat water to feed boilers, and energy in the form of heat which would otherwise be wasted is employed to do useful work. Assuming that every care is taken in the installation of steam plant to ensure that the greatest possible use is made of the steam generated, and that all fittings of every description are of the best and most efficient type, the greatest care and watchfulness is required in working to check waste and to economise the upkeep. It should be possible by means of water meters and by measuring both coal and the resultant ashes to ascertain accurately whether the right output of steam is being got from the coal consumed. Add to this periodical and thorough examination of boilers and their fittings, and of all apparatus in connection with them, and the question of cost of upkeep can be reduced to its lowest point consistently with efficiency.

In this very rough outline of the engineering work of a hospital, many important details have perforce been omitted—the point I wish to enforce being the importance of careful design on the one hand and the most perfect construction on the other as the chief factors in reducing the cost of upkeep.

The same principles will be found to apply to the important subject of sanitary fittings and drainage. It is hardly necessary in these days to emphasise the need for periodical examination and testing of the drainage system; but the regular examination of sanitary fittings and of such details as taps and valves is not perhaps so universally carried out. Nevertheless, it is of great importance that these things should be systematically examined. Waste of water is wrong, and is very rightly guarded against by public authorities; but when, as is frequently the case, water has to be paid for by measure, waste means avoidable cost of upkeep.

The constantly recurring necessity for repairing water taps and valves is due to the fact that extraneous matter carried by the water attacks the seatings and causes leaks. In all hospitals of sufficient size to warrant the employment of a skilled engineer, it will be found a sound economy to supply him with a lathe. Besides turning the seatings of valves, it will enable him to carry out a host of other repairs at little or no cost, and the saving effected will in a very short time cover the outlay.

An important point in connection with the plumbing fittings is that stop valves should be fitted to all branch service pipes. By doing this, taps can be renewed to any fitting without putting out of use any but the one fitting affected.

The stopping-up of waste-pipes is a constantly recurring difficulty that has to be promptly dealt with, and is generally, if not invariably, the result of carelessness. House flannel, brushes, dusters, cotton-wool, and dead flowers are among the most constant causes of stoppage, and as it would be too much to hope that these things will not be deposited in sink or other waste-pipes, it is necessary to facilitate the process of localising and removing the obstacle. For this purpose all waste and soil pipes should be provided with cleaning eyes with screw caps in readily accessible positions.

Details of this kind and such things as stop valves all tend to add to the initial cost, but this expense will be saved over and over again in the long run.

All hospitals should be supplied with suitable appliances for dealing with an outbreak of fire, and these things should be periodically examined by a competent person and a report made to the governing body. It is always advisable to seek the advice of the public authority which is concerned with fires (*e.g.* in London the L.C.C. Fire Brigade) before deciding on the kind of apparatus to be installed. Much money may be wasted in providing a costly system of hydrants when a sufficient number of hand-apparatus would have been of more practical use. The regular systematic instruction of the staff in the use of the apparatus is a matter of first importance. It is hardly within my province to discuss details of electric lighting or power work, but there are some points to which I may, without impropriety, allude. There is, first, the important subject of economy, and there is hardly anything in which saving is so easy and is so seldom practised as in electric lighting. It is so simple to turn off the light when a room is vacated, if only for a few minutes, and so few people ever do it. At the London Hospital each switch is inscribed "Please do not waste," and this appeal is said to be fairly effectual.

I suppose all engineers would agree that the best system of wiring is that of screwed tubing buried in the walls, with draw-boxes at suitable points. On all distributingboards each fuse should be numbered, and the various circuits numbered to correspond. This facilitates and simplifies the locating of faults.

The arrangement of switches requires careful working out; it is as a rule false economy to put three lights on to one switch, and in most cases three switches would be better than two.

In wards a plug or a bracket, or both, should be provided to each bed. A bracket which can be unhooked and used as a hand-lamp is probably the most convenient arrangement. For general lighting of the wards, the ideal system to my mind is by pendants with inverted shades, which reflect the light on to the ceiling and shade the lamps from the patients' eyes; and by a parallel series arrangement of the wiring the light can be dimmed down for night use without wasting current by inserting a resistance.

For lighting sanitary offices, automatic door-switches controlled by a switch outside are useful in saving current.

By the introduction of automatically-controlled electric lifts, a substantial saving is possible by the entire elimination of the lift attendant.

We can hardly leave the subject of upkeep of hospitals without some reference to the question of the way repairs should be carried out. Without going into the rather difficult question of whether a hospital ought to have a works' department or not, and, if so, to what extent it should be carried, I think that in all except the smallest class of hospital, some arrangements should be made for carrying out repairs to electric-light work, plumbing and engineering, and, to a limited extent, painting. At the London Fever Hospital, with which I have been connected for many years, our engineer has under him a handyman, who carries out innumerable repairs to furniture and fittings, repairs glass, and carries out small painting jobs. The engineer does all minor repairs to the machinery and the electric-lighting plant and fittings, and for simple repairs or alterations he is frequently authorised to employ a bricklayer or plumber from outside to work under his supervision. A most useful part of the work done under the engineer is the preparation of the material used in polishing the ward floors. Nearly all our floors are of polished teak, and I found some years ago that we were spending a large sum yearly in buying a ready-made preparation. I ascertained exactly what the constituent parts of this were, and found that by buying the materials and making our own polish we were able to save a very large sum annually.

The annual cleaning of wards or extensive works of painting cannot, I think, be undertaken economically by the employment of direct labour. Work of this kind, to be thoroughly well done, involves the employment of a skilled staff, and cannot be efficiently or economically carried out by the aid of a scratch team of men got together for the purpose. In painting, perhaps more than any other trade, the quality of the labour is of vastly more importance than the material used. By going to a manufacturer of established reputation and buying the best materials you have a reliable guarantee of their quality. But the best material may be ruined by want of skill on the part of the workman.

In this brief summary of the subject of upkeep of hospitals I have endeavoured to touch on the most important points without entering into unnecessary detail; and if perchance it may be thought that I have condensed too much, I hope that at least I have provided material for fruitful and useful discussion.

### THE DISCUSSION ON THE PAPER.

Mr. Conrad Thies, Honorary Secretary, British Hospitals Association,

Said that he invited Mr. Keith Young to contribute this paper, feeling how useful it would be to have an authoritative presentment on such a practical subject from an experienced hospital architect. He (the speaker) looked back upon some twenty-five years' experience in connection with the work of a general hospital. Some of the most difficult problems he had had to solve were connected with the upkeep of the various departments of his hospital. Hospital secretaries had not usually received any training in building, drainage, engineering, etc., all of which subjects were of the utmost importance in respect of the efficient and economical upkeep of an institution. He had found it necessary, therefore, to study many subjects of which he had previously been entirely ignorant and to obtain practical advice from experts. It had formerly been the custom at his hospital for two or three members of the Board to go round the hospital once in each year to inspect the wards and various departments and decide whether they needed renovating. But in course of time a better system was pursued, and each ward, etc., received due renovation at regular intervals, whatever its casual appearance might be. In this way the whole of the institution was maintained in a thorough state of efficiency. A subject that had exercised his mind a good deal was the important question of engineering; on that aspect of this question he hoped that one or two of the experts who were present would give the meeting the benefit of their views. At the Royal Free Hospital, with which he had been officially connected, over £80,000 had been expended during the past twenty-five years in alterations, additions, and improvements, including new buildings and additions, also the installation of modern systems of drainage, engineering, and electric lighting. Lack of practical knowledge on these matters, sometimes even when experts had been consulted, had caused waste of money and loss of efficiency. In his opinion it was essential that the best expert advice should be obtained. It was exceedingly difficult to avoid making mistakes. Even in the latest constructed large hospital in London he saw some things which seemed likely to cause trouble and expense in the future. In connection with the recent important additions and improvements to St. Bartholomew's Hospital and its elaborate new out-patients' department, he was surprised to find that there were no facilities for wheeling an ambulance directly from the street into the casualty receiving room, so that the patient had to be carried up some steps, which surely was a serious oversight. He desired to thank Mr. Keith Young for his address, and he hoped those present would carry the discussion to a practical issue.

Mr. Eason, Children's Hospital, Manchester,

Commented on Mr. Young's reference to a "scratch team of painters," and said that in many hospitals there was what could be termed a works department, and they were competent men, and worked under the supervision of the hospital architect. He thought the method must effect a considerable saving.

Mr. H. J. Collins, House Governor, General Hospital, Birmingham,

Said his own institution was now sixteen years old, but all concerned with the upkeep of hospitals must be impressed with two points in the paper : the cleansing eyes, or caps, for the cleansing of drains, and the stop-taps. Having started with very few of those, and having added them from time to time, he had realised the importance of accessibility of each small section, and the necessity of cutting off each section so that a tap could be repaired without much inconvenience. He had no works department in his hospital, but he had a very practical engineer, who had a fitter and a boy, and they did the ordinary repairs about the hospital as well as supervising the machinery for the ventilators. Very little plumbing was necessary, as most of the pipes were of iron. The other repairs were done by a carpenter and joiner. The ceilings and walls of the wards should be coated with paint enamel, not whitewashed and distempered. Years ago he worked out the comparative cost, and found it to be in favour of the enamel, notwithstanding the heavier first cost of the latter. Moreover, there was in that way a considerable saving in light throughout the year. The walls were thoroughly washed, in addition, as often as necessary. Moreover, distemper held the moisture and the dust. He would like to hear Mr. Young speak, in his reply, as to the condensation occurring on glazed brick and tile, and therefore the aggregation of germs. If there was no liability to such, then, in erecting hospitals, glazed brick and tile would have a preference over surfaces which required to be kept painted or whitewashed.

Mr. Smith, Children's Hospital, Bristol,

Would like to know how often it was desirable to have the sanitary work of a hospital inspected. At his hospital a sanitary inspector from the Public Health Department went round with the contractor once a year, which might be considered as somewhat too frequent. He hoped the author of the paper would give some valuable information as to the kind of fuel to use in the heating apparatus. Was it more economical to use coke alone, or anthracite coal alone, or a combination of the two?

Mr. J. S. Neil, Wolverhampton,

Pointed out that for the majority of hospitals already built it would be impossible to convert the present windows to those with a reversible sash. He would like the point discussed whether the window cleaning should be done by the hospital porters, or by an outside firm. His experience was that the latter paid the better. Drainage was most important, and the whole success of a hospital rested upon it. Lead pipes should be abandoned, and only iron used, for the former easily buckled and caused trouble. Inspection-eyes should be placed at frequent intervals. A little forethought might often have obviated carrying pipes round corners, and every hospital should have a supply of drainage rods. Perhaps the men who were employed to do the work about the hospital could be called a "scratch team," but, if so, they were an efficient one. He agreed with Mr. Collins that in the long run enamel was cheaper than colour-washing, and that applied to ceilings as well as walls. In many parts of a hospital he did not think it necessary to go to the expense of glazed bricks and tiles, especially as, under certain atmospheric conditions, they were liable to sweat. Good cement served equally well. With regard to fuel, if the boilers were put in by a competent man, and had a suitable size of flue, there was no need for expensive fuel; slack could be efficiently burned, and was cheaper than coal. Colliers always burned up their worst rubbish, but that was not practicable in a London hospital owing to restriction of space. The heating of wards was done cheapest by means of low-pressure hot water, heated by calorifiers; steam could be used for the laundry and for sterilising in the laboratory. He did not think an efficient hot-water service for taps could be secured without an accelerating system. With regard to flooring, terrazzo invariably cracked, and it had not a good effect on the nurses in the wards, as it was cold and hard to the feet, and he had noticed nurses develop flat-foot on it. Linoleum was nice, but did not wear as well as teak blocks, which latter he regarded as in many ways ideal. If laid on plastic material, the joints were very small, and the blocks could not give way. He did not see why an operating theatre should not be erected without any projection in it, all pipes being directed to, and attended to, in a double bay, without requiring the workmen to enter the theatre. Also, instead of having the sinks built into the floor or wall, a floor socket could be made and a pillar tightly fitting into that, and the sinks laid on a cross-piece. That enabled the sink to be taken away in five minutes without disturbing or damaging the floor or wall. And the hot-water, drainage, and other pipes should be built into the floor box; and similarly with the electric fittings.

---

**(Continuation of Discussion.)**

Mr. Griffith, East Suffolk,

Expressed his agreement with the remarks of Mr. Thies. The paper was an excellent one, but it touched chiefly those about to build or about to enlarge or alter structures. He agreed that the window-cleaning was better done by outside contractors; in the case of his own hospital the porters could not do it, because the windows were too lofty. He advocated the use of governors supplementary to gas meters, as they effected great economy. In regard to electric lighting, he counselled the rejection of all carbon-filament lamps, and the installation of those with metal filaments, those which would not consume more than 1 watt per candle. Much trouble had been caused by the local sanitary inspectors and surveyors not properly knowing their work. He wished to congratulate the authorities at the Radcliffe Hospital on their new boiler; running one huge boiler and using calorifiers was the cheapest system. The best reversible sash for hospital windows was the "Austral." For flooring he preferred "Ednorite." He expressed his indebtedness to Mr. Thies for the seasonable help he had several times afforded him.

Sir Henry Burdett

Expressed his warm appreciation of the practical character of the paper. In early days it was difficult to find men in charge of hospitals who were able to understand the various matters which had just been discussed. It must be clear to any listener that the present managers of hospitals had given their time and study to everything appertaining to their work. Mr. Thies had called attention to a serious defect in the casualty department of a London hospital which had cost £80,000, where the patient had to be carried up some steps, and so the risk was run of converting a simple fracture into a compound or comminuted one. The great need when constructing or altering a hospital was to have a consultation between the architect and those who would be called upon to work the hospital. And when once the plan and system had been decided upon, no subsequent interference with the plans during erection should be permitted; moreover, such interference invariably greatly added to the expense. In the case of the Manchester Royal Infirmary the Board wisely appointed a capable superintendent before the work of building was begun, and that institution showed sound practical knowledge right through, and Mr. Carnt secured a way of doing things which was almost unique. A different procedure was followed at the Royal Victoria Hospital, Newcastle, and the result was also different. When in the States, he noticed that Dr. Washburn, now the medical superintendent of the Massachusetts General Hospital, had reduced the control of the repairs and the maintenance of structural efficiency to a remarkable system. At Newcastle Royal Infirmary Mr. Ord had now organised a wonderfully efficient team in all his departments. To many members of so-called "scratch teams" hospitals were indebted for a number of useful devices, and he was anxious that the artisans and craftsmen working in our hospitals should be encouraged. To Mr. Young the Association must feel greatly indebted, for he had done much to promote efficiency in the hospitals which passed through his hands.

Mr. Carnt, Manchester Royal Infirmary,

Pointed out that in the case of some of the engineering details it was sometimes wisest to accept the highest tender, for it meant greater ultimate economy and safety. That was done at his institution, with the result that 400 people were cooked for at an expenditure of 2s. 3d. a day for gas. Later, 850 people were cooked for at an outlay in gas of only 5s. 9d. For electric light and treatment he strongly advocated the installation of a series of sub-meters; it enabled any leakage to be detected, and the results of the readings in the different wards revealed a healthy rivalry in the matter of economy. Water-meters were also desirable. Careful checking and reorganisation of the electric supply from the Corporation brought the charge for electricity down from £280 to £84. The substitution of steam for heating in place of gas had also produced a great saving. Window cleaning at his infirmary was done by the staff of twenty-three porters. The attendances of the medical staff were put in in the mornings, and hence the porters were left free in the afternoon for other work. The perfect hospital floor had not yet been discovered. At his hospital £3,500 worth of linoleum had been laid down, and he was better pleased with it than he thought he would be. He lived for fourteen years in a hospital which had terrazzo floors, but heard of none of the disadvantages from it which had been described, and he had not heard of a case of flat-foot in any of the nurses in his institutions during twenty-two years of hospital management.

Mr. Bray, General Infirmary, Leeds,

Expressed his high appreciation of the paper. His experience of hospital architects was that they generally went into details in their work in a very thorough manner. At his institution a good deal of gas, electric light and water had been saved by installing a number of meters or sub-meters at different points. On one occasion a leakage of 8,000 gallons per day was detected by the meter; there was no surface indication of it, as the hospital stood on a slope. He did not understand Mr. Young to mean by "scratch team" the men usually kept on the premises to do the repairs; employing outside casual men sometimes resulted in getting men whose work was inexperienced and wasteful. If there were a definite works department at a hospital there was a liability, in order to find the men something to do, to undertake work which otherwise would not have been touched. The window-cleaning arrangement would largely depend on the height and size of the windows at each particular place. In regard to the walls, his opinion was opposed to Mr. Collins. Seven of his sixteen wards were tiled to a height of 6 feet, and they were colour-washed above. Leeds had a dirty atmosphere, and at his hospital there was a thorough cleansing once a year or oftener. The kind of fuel to use for the boilers depended on the neighbourhood one was in; but for economy hard washed nuts were best. If there was a regular demand throughout the twenty-four hours, one could do very well with slack, though it caused great dust and smuts from the chimney, which got into the wards. For flooring he had a strong preference for teak boards 4 inches wide. If well waxed the joints were practically non-existent.

Mr. Walrond, Great Ormond Street Hospital, London,

Said he thought drains should be subjected to a severe test, with a standpipe from a height of 25 feet, and subsequently with the air test. The smoke test was negative in value. For the drains there should be iron inspection-chambers; then after testing with air and finding a defect it was not necessary to open a manhole. He tested the drains and soil pipes of the Hospital for Sick Children, Great Ormond Street, London, for seven years twice a year. In some cases it was necessary to flush not only the drains but also the tanks. It would be surprising to some to see how much deposit had formed. He advocated the use of exhaust steam for heating purposes. With regard to fuel, in a large boiler the use of coke would be found to be expensive. For anthracite there must be a good draught. In London the authorities did not allow one to use rubbish, because of the smoke nuisance. He regarded a steel tube for the electric wire as an unnecessary expense; moreover, moisture condensed on it to such an extent as to ruin the wiring in a few years. The casing should be put out of the way, as near the ceiling as possible. He also pleaded for the use of automatic telephones.

Dr. Mackintosh, Glasgow,

Remarked that hospital managers were only too willing to co-operate with the architects, but no hospital manager would seek to interfere with the architect's designs. He had derived much benefit from the discussion. The question of gas, electric light, etc., must be decided on its merits in each individual case. To get the best results there must be a combination of competent architect and a man at the helm who could run the hospital efficiently and economically.

Mr. Keith Young,

In his reply, explained that he applied the term "scratch team" merely to the painters. By picking up chance men for that, one got bad and uneconomical work, and it was better to employ an outside firm who would do the work well. It had been found that the tiled bricks gave the reverse of condensation, and they saved enormously on the annual cleaning bill. Slack, even when burned in high-pressure boilers, proved a nuisance to the wards, and sometimes to the neighbourhood. Block floors he regarded as bad. Screwed tubes with an insulated coating inside for electric wires showed no condensation anywhere; there had been a good trial of them at the Middlesex Hospital. He expressed his pleasure at hearing the discussion and the courtesy shown to him.

**Thanks to Sir William Osler.**

The President having to leave early, Dr. Mackintosh proposed a very cordial vote of thanks to him for his kindness, and it was carried by acclamation.

In reply, Sir Wm. Osler said that any reputation he had acquired had been made by hospitals, as perhaps no man in the profession had lived in hospitals more than he had. Members of that Association were the men the profession looked up to to carry out the new ideas and ideals. He directed attention to the ideal hospital among English-speaking people, the Peter Brigham Hospital in Boston, U.S.A., full information of which would be sent by the superintendent of that institution. There they had built only two units, a medical and a surgical. The other portions of the work were developed in the neighbourhood, so there was no reduplication of the work. The plan appealed to one by its simplicity and by the facility in work which was made possible. There was but a single head in each department, having a group of accredited assistants working with him in the wards and directing the laboratories. The hospital authorities had provided the head with a suite of rooms for his consultation work, and they paid him a good salary. And hospital men should bear in mind that they will be asked in the future to build hospitals for university purposes on lines which had not hitherto been carried out.

### THE BUSINESS MEETING.

At the business meeting held on Friday, June 27, at the close of the Conference, Dr. Mackintosh in the chair, a cordial vote of thanks was accorded to the Vice-Chancellor of the University for his kindness in allowing the meetings to be held in the Examination Schools.

Thanks were also accorded to Sir William Osler, Bart., D.M., for presiding at the Conference and to Sir Thomas Oliver and Mr. Keith Young for contributing papers for discussion.

Upon the motion of the Chairman, Sir William Osler was unanimously elected President of the Association for the ensuing year.

The Chairman reported that Sir Henry Burdett had tendered his resignation of his seat on the Council, which had been accepted with regret, and it was resolved unanimously: "That Sir Henry C. Burdett, K.C.B., K.C.V.O., be elected a Vice-President in recognition of the invaluable services he had rendered to the Association."

It was also resolved unanimously: "That Sir William Cobbett be invited to become a Vice-President in recognition of his services as President of the Manchester Conference."

A vote of thanks was given to Mr. Arthur F. Whinney for his kindness in auditing the accounts of the Association, and he was unanimously elected Honorary Auditor for the ensuing year.

Mr. E. Stewart Johnson and Mr. R. A. Outhwaite were elected Honorary Treasurers, and Mr. Conrad W. Thies and Mr. Alexander Hayes, Honorary Secretaries.

All the members of the Council were re-elected with the exception of Sir Henry Burdett, Mr. Albert Cay, and Dr. Friend, who had expressed their desire to retire; and the undermentioned gentlemen were elected to vacant seats:—Mr. W. K. Alvey, Charing Cross Hospital; Mr. Allan E. Batchelor, Warneford Hospital, Leamington; Mr. George Priestman; Mr. Percy Somers, Cottage Hospital, Bushey; Dr. Nathan Raw, Mill Road Infirmary, Liverpool.

On Friday afternoon a visit was paid to Nuneham Park by river, by the courtesy of the Right Hon. L. Harcourt, Sir William Osler and other gentlemen kindly providing a steam launch.

